# Supplementary material for: The effect of levocarnitine supplementation on dialysis-related hypotension: A systematic review, meta-analysis, and trial sequential analysis
Source: PLoS One. 2022 Jul 14;17(7):e0271307. doi: 10.1371/journal.pone.0271307 (PMC9282471; doi:10.1371/journal.pone.0271307)
Supplement: S2 File — (DOCX) [file pone.0271307.s002.docx]

**Supplementary Material**

The following MEDLINE subject headings (MeSH) search terms were used to search for trials within MEDLINE (PUBMED):

((Levocarnitine[tiab] OR l-carnitine[tiab] OR "carnitine"[MeSH] OR Carnitine[tiab]) AND (Dialysis[tiab] OR Hemodialysis[tiab] OR kidney failure[tiab] OR “End-Stage Renal Disease” OR ESRD OR "Dialysis"[Mesh] OR "Renal Dialysis"[Mesh] OR "Dialysis Solutions"[Mesh] OR "Kidneys, Artificial"[Mesh] OR "Hybrid Renal Replacement Therapy"[Mesh] OR "Hemodialysis Solutions"[Mesh] OR "Continuous Renal Replacement Therapy"[Mesh] OR "Renal Insufficiency"[Mesh] OR "Kidney Failure, Chronic"[Mesh] OR "Acute Kidney Injury"[Mesh])) AND (Hypotensi*[tiab] OR intradialytic hypotension[tiab] OR cardiac function[tiab] OR "Hypotension"[Mesh] OR low blood pressure[tiab])

The same search strategy was used for EMBASE and Cochrane Central Register of Controlled Trials using the corresponding terms. Below is the search strategy for EMBASE:

#1: levocarnitine:ab,ti OR 'l-carnitine':ab,ti OR 'carnitine'/exp OR carnitine:ab,ti

#2: dialysis:ab,ti OR hemodialysis:ab,ti OR 'kidney failure':ab,ti OR 'end-stage renal disease' OR esrd OR 'dialysis'/exp OR 'renal replacement therapy'/exp OR 'hemodialysis'/exp OR 'dialysis fluid'/exp OR 'dialyzer'/exp OR 'kidney failure'/exp OR 'chronic kidney failure'/exp OR 'acute kidney failure'/exp

#3: #1 AND #2

#4: hypotensi*:ab,ti OR 'intradialytic hypotension':ab,ti OR 'cardiac function':ab,ti OR 'hypotension'/exp OR 'low blood pressure':ab,ti
#5: #3 AND #4
